# Cost-effectiveness analysis of surgical proximal femur fracture prevention in elderly: a Markov cohort simulation model

**DOI:** 10.1186/s12962-023-00482-4

**Published:** 2023-10-25

**Authors:** Momin S. Alnemer, Konstantin E. Kotliar, Valentin Neuhaus, Hans-Christoph Pape, Bernhard D. Ciritsis

**Affiliations:** 1 Department of Medical Engineering and Technomathematics, Aachen University of Applied Sciences, Campus Juelich, Heinrich-Mussmann-Str. 1, 52428 Juelich, Germany; 2 Trauma Surgery Unit, Universitätsspital Zürich, Rämistrasse 100, Zürich, 8091 Switzerland; 3 Orthopaedic Surgery Unit, Centro Ortopedico di Quadrante, Lungolago Buozzi, 25, Omegna, VB 28887 Italy

**Keywords:** Hip fractures, Prevention, Geriatric, Cost-effectiveness, Prophylaxis, Healthcare utilization, Quality-adjusted life-year, Finite-State Markov Model, Sensitivity analysis, Surgical Prophylaxis

## Abstract

**Background:**

Hip fractures are a common and costly health problem, resulting in significant morbidity and mortality, as well as high costs for healthcare systems, especially for the elderly. Implementing surgical preventive strategies has the potential to improve the quality of life and reduce the burden on healthcare resources, particularly in the long term. However, there are currently limited guidelines for standardizing hip fracture prophylaxis practices.

**Methods:**

This study used a cost-effectiveness analysis with a finite-state Markov model and cohort simulation to evaluate the primary and secondary surgical prevention of hip fractures in the elderly. Patients aged 60 to 90 years were simulated in two different models (A and B) to assess prevention at different levels. Model A assumed prophylaxis was performed during the fracture operation on the contralateral side, while Model B included individuals with high fracture risk factors. Costs were obtained from the Centers for Medicare & Medicaid Services, and transition probabilities and health state utilities were derived from available literature. The baseline assumption was a 10% reduction in fracture risk after prophylaxis. A sensitivity analysis was also conducted to assess the reliability and variability of the results.

**Results:**

With a 10% fracture risk reduction, model A costs between $8,850 and $46,940 per quality-adjusted life-year ($/QALY). Additionally, it proved most cost-effective in the age range between 61 and 81 years. The sensitivity analysis established that a reduction of ≥ 2.8% is needed for prophylaxis to be definitely cost-effective. The cost-effectiveness at the secondary prevention level was most sensitive to the cost of the contralateral side’s prophylaxis, the patient’s age, and fracture treatment cost. For high-risk patients with no fracture history, the cost-effectiveness of a preventive strategy depends on their risk profile. In the baseline analysis, the incremental cost-effectiveness ratio at the primary prevention level varied between $11,000/QALY and $74,000/QALY, which is below the defined willingness to pay threshold.

**Conclusion:**

Due to the high cost of hip fracture treatment and its increased morbidity, surgical prophylaxis strategies have demonstrated that they can significantly relieve the healthcare system. Various key assumptions facilitated the modeling, allowing for adequate room for uncertainty. Further research is needed to evaluate health-state-associated risks.

**Supplementary Information:**

The online version contains supplementary material available at 10.1186/s12962-023-00482-4.

## Background

One of the most vital social transformations of the twenty-first century is the aging of the population. According to data from World Population Prospects: the 2019 Revision, by 2050, one in four persons living in Europe and Northern America could be aged 65 or over [1, 2]. Population aging poses many challenges, as it is crucial for the health care system to adapt to these demographic changes. As a consequence of population aging, from 2005 to 2025, a 50% increase in the number of fragility fractures is predicted [3]. In general, fragility fractures are a socioeconomic burden, particularly hip fractures [4–8]. Initially, a hip fracture reduces life expectancy, depending on the patient’s age, by an average of 1.8 years [9]. Furthermore, only 50% of patients regain their prefracture mobility [10]. A year after the fracture, 40% of the injured individuals are still incapable of walking without support, 60% struggle with at least one essential daily life activity, and 80% are restricted in other activities [11]. Moreover, 27% of hip fracture patients enter a nursing home for the first time [11].

These challenges cause a burden on the individual and significant economic liability. In 2005, hip fractures accounted for only 14% of the total incident fractures and necessitated 72% of fractures’ total cost [11]. The number of hip fractures is projected to increase from 1.26 million in 1990 to 4.5 million in 2050 [12]. That increase will result in a rise in hip fractures’ total cost up to 95 billion US dollars ($), assuming that one patient’s hip fracture repair costs just $21,000 [10]. Taking into account that this study was conducted in 1997, the cost in today’s money is approximately $34,000 per patient. Making allowances for inflation and different factors, another paper by Frick et al. estimated the average societal cost to be much higher $86,967 ± 21,225 (mean ± std. deviation) [13]. Based on this estimate, if no new intervention is developed, the economic magnitude of hip fractures in the year 2050 will be approximately 392 billion US dollars.

The statistics mentioned above and the literature on hip fractures show the severe pressure caused by them on the health care system [4–11, 14, 15]. With that in mind, if a new surgical intervention that decreases fracture risk is developed, it is very likely to be an impactful relief for the health care system [13, 16, 17]. Today, a couple of prophylactic treatments that help decrease the probability of a hip fracture are being studied. Nevertheless, they are associated with increased morbidity and might cause future complications [18]. A promising approach is the prophylactic augmentation of the proximal femur with composite bone cement, mostly poly(methyl methacrylate) (PMMA). Analogous to the well-established cement augmentation of spinal bodies, vertebroplasty and kyphoplasty, this approach is known as femoroplasty. In recent decades, numerous studies have been conducted using a wide range of bone cement to assess the effectiveness of femoroplasty. All of them proved that it could significantly increase the stability and strength of the proximal femur without affecting the stiffness. Whether as support after or before fracture fixation or as a prophylactic procedure [18–26]. In vitro, femoroplasty proved that it could increase the work to fracture by 33.2% and the fracture force by 19.9% for pertrochanteric and femoral neck fractures using standardized and reproducible techniques and instrumentation [19]. However, clear guidelines to support decision-makers on when to follow a preventive strategy are limited in today’s literature.

Various well-established statistical approaches help decision-makers incorporate strategies that benefit the most from available resources. Performing a cost-effectiveness analysis (CEA) identifies medical techniques to realize the most significant health gains [27]. Accordingly, implementing a more cost-effective strategy is as good as acquiring new resources. Inevitably, an orthopedic surgeon should make a subjective judgment based on the presented facts. Clinical decision support systems better grasp optimal decisions and aid treatment judgments. This study aims to identify patient-related factors for which a surgical orthopedic preventive strategy (femoroplasty) would be cost-effective at either the primary or secondary prevention level. Markov modeling is a long-established method to determine the cost-effectiveness of medical intervention strategies [27–30]. Since costs and health states are well defined in the hip fracture literature, we created a finite-state Markov model to perform a cost-effectiveness analysis.

## Materials and methods

### Model

**Fig. 1:**
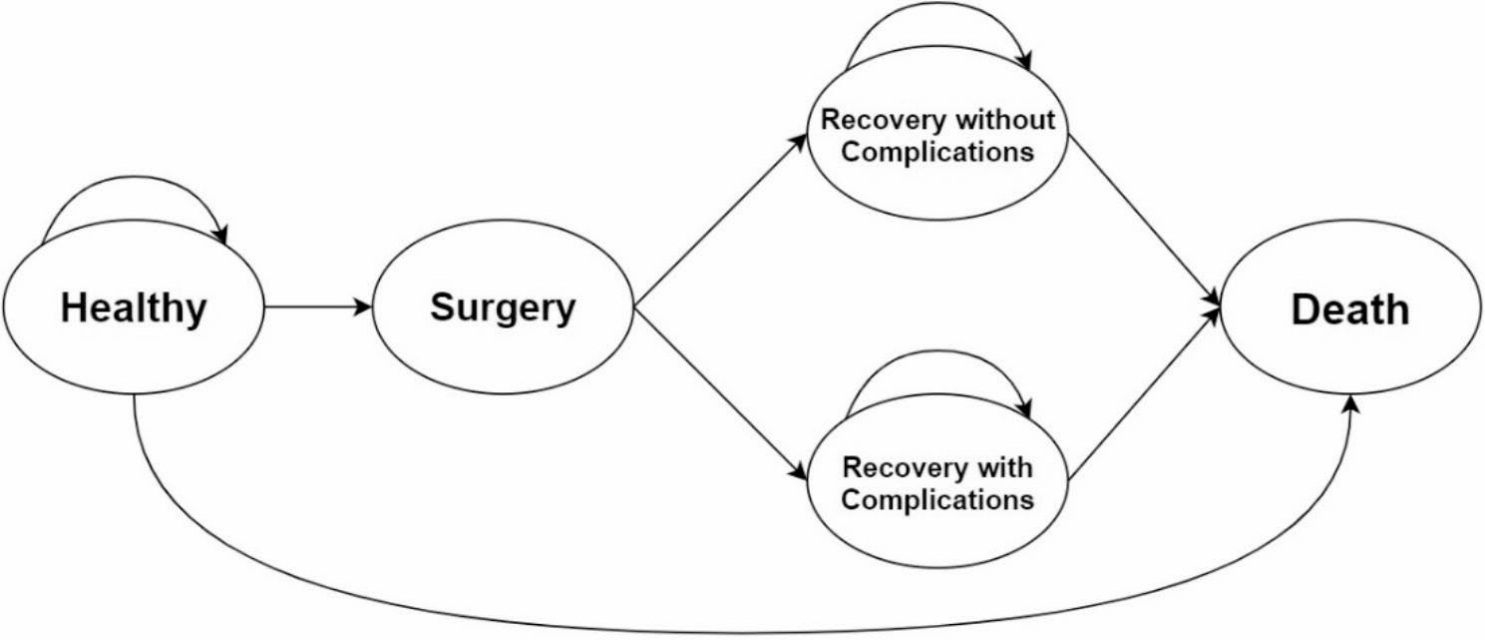
Model design for models A & B showing the health states (Ellipses) and the transition probabilities (Arrows). A cohort of identical individuals enters the Markov model and transitions through different health states with their correlated costs and QALYs

We created a model with cohort simulation, which means that a cohort of identical individuals was simulated and had during any model cycle to be in one of the defined health states [28] (Healthy, Recovery with complications, Recovery without complications, and Death) (Fig. 1). The model is from a societal perspective with a lifetime time horizon. A decision tree with Markov nodes can be found within the supplementary material 1 accompanying the manuscript. Due to the availability of literature, the model is based on data from the United States. However, it can apply comparatively to any industrial country with a high life expectancy. The model’s cycle length was defined as one year. Complications are modeled to occur within one cycle of the initial fracture, and the surgery’s length is less than one cycle. Therefore, the health state “Surgery” is a transitional health state, which denotes that individuals in this health state never stay in it for a complete cycle. A CEA has three core components: costs, utilities, and transition probabilities. Costs were calculated in United States dollars (US$). Utilities allow the comparison of health effects and changes in quality of life. We measured them as quality-adjusted life years (QALYs).

The result of a CEA is the additional investment of resources needed for each additional unit of health improvement expected to result from investing in the new treatment. It is the incremental cost-effectiveness ratio (ICER) with the unit dollars per QALY ($/QALY). $50,000 - $100,000/QALY is the most common range for the willingness to pay (WTP) [31]. WTP is the threshold for the ICER, and we defined it as $100,000/QALY. The WHO advised that the WTP should be one to three times the country’s GDP per capita [29]. Hence, we considered an ICER less than $50,000/QALY definitely cost-effective and an ICER less than $100,000/QALY likely cost-effective. Calculations were performed using TreeAge software (TreeAge Pro Healthcare 2020, TreeAge Software, LLC, Massachusetts, USA).

A CEA using a Markov model is commonly employed in medicine to compare two treatment strategies [32]. This study compares the current medical practices without prevention treatments with a strategy where a low-cost mini-invasive prophylactic procedure (femoroplasty) is steadily employed. Two key target groups for which such a scenario could be cost-effective were defined. Target Group 1: Patients who already suffered from a femoral neck fracture on one side as they have a 5- to 10-fold higher risk of breaking the contralateral side [33–35]. Target Group 2: Patients presented with high fracture risk factors, such as osteoporosis (T-score of -2.5), glucocorticoids, tobacco and alcohol use, female sex, or parent fractured hip but with no fracture history. We carried out a CEA that evaluated only these two target groups. To do so, we created two models, model A, in which we assumed that 100% of the healthy population had already suffered from a fracture in the hip. Model B assumes that all individuals who enter the model belong to a high fracture risk group. Although both models have the same structure (Fig. 1), they differ in costs, transition probabilities, and utility scores. The baseline patient age was 60 years, and we also assessed patients aged 60 to 90 years, as they are most prone to hip fractures.

### Transition probabilities

Transition probability is the likelihood of transitioning from one health state to another. We acquired the age-dependent model probabilities from the available literature, as shown in Table 1. Given that the literature scope is limited for older age groups, transition probabilities for ages over 90 years were extrapolated from past trends assuming linearity. An exception is the general all-cause mortality rate, which we adapted online for all ages from a website that calculated it for life insurance purposes [36]. All probabilities were considered equal with and without prophylaxis, except for the risk of sustaining a hip fracture. Due to the mentioned biomechanical findings, it is safe to assume that surgical prophylaxis will reduce fracture probability [19]. Therefore, we predicted a 10% relative reduction in fracture probability in the baseline analysis due to prophylactic surgery. This assumption is based on expert approximation, as it is impossible to translate biomechanical values into probabilities. Different percentages from 1 to 50% were also assessed in the sensitivity analysis.

**Table 1:** Model’s age-dependent transition probabilities. The low and high parameter ranges were estimated as 60–120% of the base value shown

Age	60	70	90	Source
All-cause mortality rate	1.14%	2.34%	16.45%	[36]
Probability of contralateral hip fracture (model A primary analysis)	4.13%	5.09%	6.36%	[16]
Probability of contralateral hip fracture (model A secondary analysis)	0.41%	0.61%	0.74%	[37]
Probability of contralateral hip fracture (model A tertiary analysis)	0.44%	1.66%	4.41%	[17]
Probability of hip fracture in high risk (model B primary analysis)	0.68%	3.93%	10.23%	[37]
Probability of hip fracture in high risk (model B secondary analysis)	0.04%	0.22%	0.93%	[17]
Probability of complications	17.00%	26.00%	40.00%	[6, 16, 38]

For model A, the contralateral fracture probability was acquired from three sources [16, 17, 37]. As every source considers different factors to estimate this probability, we performed the calculations three times using each source. Regarding model B, we used two references that estimated the probability of the first hip fracture for a high-risk group [17, 37]. The FRAX® [37] tool was used as one of the sources for both models. FRAX® is a prognostic tool created by the WHO to preview the individual risk of femoral neck fracture in 10 years. When calculating this risk from the FRAX® [37] tool, we used the USA’s average body mass index (BMI) of 29.6 [39]. A T-score of -2.5 was incorporated during the calculations. The risk factors assumed for model B were parent fractured hip, current smoking, glucocorticoids, rheumatoid arthritis, secondary osteoporosis, and heavy alcohol use. When calculating the one-year risk, we presumed a linear probability distribution. Supplementary pharmacologic fracture prevention therapy was disregarded in both models.

Implementing all types of preoperative complications in the model would cause it to be inexplicable and may reduce its accuracy. Therefore, we assumed that simulated patients could only sustain one preoperative complication regardless of its type. An article from Roche et al. followed 2448 hip fracture patients for four years. It concluded that 498 patients developed complications, representing approximately 20% of the total study subjects [38]. Alternatively, another study conducted by Pugely et al. followed 4331 patients undergoing hip fracture surgery. It established a total complication rate of 30% [6]. We used both articles and the article by Jiang et al. [16] to estimate the age-dependent risk of complications. After sustaining one preoperative complication, the excess mortality rate was calculated as an average from two papers [16, 38]. It was applied only for the first five years after the fracture complication.

The study by Tosteson et al. found that even if the patient recovers without complications, the mortality rate is elevated in the first six months [8]. Consequently, we assigned an elevated mortality rate only for individuals spending their first cycle in the health state “Recovery without complications” to simulate patients who died during the surgery (Table 2). After that, the mortality rates for this health state were considered equal to the general all-cause mortality rate. For model A, we assumed that the prophylactic procedure was performed during the contralateral side’s surgery. Thus, we assigned the same elevated mortality rate to individuals in the health state “Healthy” in the first cycle in model A. We estimated the low and high parameter ranges for transition probabilities as 60–120% of the base value.

**Table 2:** Model’s fixed variables

Parameter range	Low	Base	High	Source
Additional mortality in the first year after recovery	4.82	6.28	8.2	[8]
Additional mortality after complications	1.73	2.71	6.98	[16, 38]
Risk reduction of prophylaxis	1%	10%	50%	Assumption
Discount Rate	0%	3%	8%	[29]

### Costs

We defined all costs from the payer’s perspective and discounted them by 3% per year. The discount rate was varied between 0% and 8% in the sensitivity analysis. Since the simulated cohort is older than 60, we did not consider productivity gains due to improved health. We regarded no new costs if no complications were present one year after the fracture. We based our estimates of equipment and surgeon costs on the Healthcare Common Procedure Coding System (HCPCS) code 27495 (Reinforce thigh) [40] and on the expert’s approximation. Consequently, it was assumed that all hip fractures were treated using fracture fixation rather than total hip arthroplasty with code 27130, as only proximal femur fractures in the pertrochanteric area were considered. Varying the costs in the sensitivity analysis should eliminate the difference introduced by that assumption.

In model A, we assumed the prophylactic procedure was performed during the contralateral side surgery. Therefore, the null set of model A is the surgery performed during which the prophylactic treatment was executed. This means that the additional cost of prophylaxis is only the cost of the equipment used and the cost of the extra time needed for the surgeon to perform it (Table 3). We did not assume that it would increase the length of hospitalization as it is a mini-invasive procedure with negligible effect on morbidity. Meanwhile, from the payer’s perspective, all costs were considered in model B, as the patient had to be admitted to the hospital specifically to perform the surgery (Table 3). For explicitness in model B, it was assumed that the prophylactic procedure was performed only on the most fragile proximal femur.

All other costs were collected from the American Federal Government website managed by the Centers for Medicare & Medicaid Services. It included the Inpatient Prospective Payment System (IPPS) Provider Summary for All Diagnosis-Related Groups (DRG) - FY2017 [41] and the Medicare 2020 Physician Fee Schedule [40]. We obtained hospital costs from average Medicare payments for DRG codes 481 and 482 (hip and femur procedures except major joint with or without a complication or comorbidity (CC) or major complication or comorbidity (MCC)) [41]. Facility and nonfacility fees from the Medicare 2020 Physician Fee Schedule with their respective HCPCS codes and all other costs are shown in Table 3. We derived the average cost per day for the skilled nursing facility (SNF) from a report by the U.S. Department of Health and Human Services [42]. The length of stay in the SNF for hip fracture was based on available literature and expert approximation [16, 43, 44]. The low and high parameter ranges were estimated as 66–340% of the base value obtained [16].

**Table 3:** Cost tables

Cost of fracture treatment					
Code	Description	Medicare payment ($)		Source	
**DRG 482**	Hip and femur procedures except major joint without CC/MCC	9,830		[41]	
**HCPCS 99,222**	Initial hospital care	143		[40]	
**HCPCS 27,245**	Treat thigh fracture	1,300		[40]	
**HCPCS 99,203**	Office/outpatient visit new (3-month)	122		[40]	
**HCPCS 99,213**	Office/outpatient visit new (6-month)	85		[40]	
**HCPCS 99,214**	Office/outpatient visit new (12-month)	85		[40]	
**SNF**	14-day stay	4,620		[42]	
**Total**		**16,185**			
**Extra cost of fracture complications**					
**Code**	**Description**	**Medicare payment ($)**		**Source**	
**DRG 481**	Hip and femur procedures except major joint with CC	12,340		[41]	
**DRG 482**	Hip and femur procedures except major joint without CC/MCC	9,830		[41]	
**Difference**		**2,510**			
**Cost﻿ of the contralateral side’s prophylaxis (model A)**					
**Description**	**Cost ($)**		**Source**		
**Equipment**	1,200		Assumption		
**Surgeon**	500		Expert approximation		
**Total**	**1,700**				
**Cost﻿ of the primary preventive procedure (model B)**					
**Code﻿**	**Description**	**Medicare payment ($)**		**Source**	
	Equipment	1,200		Assumption	
	Surgeon	500		Expert approximation	
**HCPCS 99,222**	Initial hospital care	143		[40]	
**HCPCS 99,203**	Office/outpatient visit new (3-month)	122		[40]	
**HCPCS 99,213**	Office/outpatient visit new (6-month)	85		[40]	
**HCPCS 99,214**	Office/outpatient visit new (12-month)	85		[40]	
**SNF**	One-day stay	330		[42]	
**Total**		**2,465**			

DRG = Diagnosis-Related Groups; CC/MCC = Complication or Comorbidity / Major Complication or Comorbidity; HCPCS = Healthcare Common Procedure Coding System; SNF = Skilled Nursing Facility; The low and high parameter ranges were estimated as 66–340% of the base value shown

### Utilities

QALY is the product of a utility score and the time spent in a health state. It combines morbidity and mortality. The utility score is a value from 0 to 1, where 1 is perfect health and 0 is death. The only difference between models A and B is the normative utility for the health state “Healthy”. The age-specific normative utilities used in model B are shown in Table 4 [17]. In model A, it was considered constant for all ages, as it is challenging to obtain age-specific utilities for hip fracture patients (Table 5) [7]. Disutility was assigned for the fracture year and each year for the first five years after fracture complication (Table 5) [16]. Naturally, the quality of life deteriorates after surgery and then improves gradually as the patient recovers. Therefore, the utility score for patients spending their first cycle in the health state “Recovery without Complications” was presumed to be 0.48 and for the cycles afterward 0.63 [7] (Table 5). No disutility was assumed after prophylaxis, as no mobility loss or pain is expected from such a procedure.

**Table 4:** Age-specific normative utilities for model B [17]

Age	Normative Utility	Low	High
60	0.754	0.603	0.829
70	0.742	0.594	0.816
80	0.702	0.562	0.772
90	0.647	0.518	0.712

**Table 5:** Model’s fixed utility/disutility scores

Parameter range	Low	Base	High	Source
Utility score for health state “Healthy” in model A	0.66	0.79	0.92	[7]
Utility score for first year after recovery without complications	0.32	0.48	0.64	[7]
Utility score after recovery without complications	0.52	0.63	0.74	[7]
Disutility for the year of fracture	-0.12	-0.27	-0.41	[16]
Disutility for each year after fracture complication	-0.13	-0.31	-0.47	[16]

### Preanalysis

Before running the models and investigating the data collected, fracture treatment costs 9.5 times the prophylaxis cost on the contralateral side (model A) and 6.6 times the prophylaxis cost as a preventive procedure (model B). Therefore, it is economically advantageous to perform a prophylactic procedure on the contralateral side on almost ten patients to prevent just one fracture. Furthermore, considering QALYs, even if the cost of fracture treatment is ignored (set to zero), prophylaxis is definitely cost-effective if it improves the QALYs by 0.034 for model A and 0.049 for model B. We included three different sources for the fracture risk in an attempt to simulate all possible scenarios.

In model A, the risks and their age distributions varied drastically between the different sources. Regarding model B, the two sources from which the probability of fracture was obtained were significantly different. Since we considered all risk factors simultaneously while using the FRAX® tool, it resulted in very high fracture risk (Table 1). Bearing in mind that we did not explicitly define the high-risk group, both sources were regarded as a range subject to the surgeon’s evaluation of the risk factors. Additionally, a sensitivity analysis was performed on both sources to assess how different probability trends affect cost-effectiveness variability. The baseline analysis was defined as a 10% reduction in fracture risk after prophylaxis in the 60-year-old simulated cohort.

## Results

### Model A

Performing prophylaxis on the contralateral side for patients who sustained a hip fracture at age 60 costs between $8,850/QALY and $46,940/QALY, assuming that the fracture risk is decreased by 10% following prophylaxis (baseline). This 10% reduction in the fracture risk due to prophylactic surgery was a sensible assumption, as higher reduction percentages caused all models to be more definitely cost-effective. However, the sensitivity analysis showed that the ICER varies significantly with variations in this reduction percentage. The sensitivity analysis proved that even if prophylactic surgery reduces the one-year fracture risk by as low as 2,8%, it may potentially be cost-effective for a 60-year-old patient (ICER less than $50,000/QALY). We additionally managed to approximate the ten-year minimum required age-dependent fracture risk reduction for prophylaxis to be definitely cost-effective in both models (Table 6). This required risk reduction fluctuated based on the baseline fracture risk and how it was spread among various age groups.

**Table 6:** Minimum required ten-year fracture risk for prophylaxis to be definitely cost-effective (ICER < $50,000/QALY). The ten-year probability was calculated while assuming a constant risk rate as it is easier to interpret than the 1-year probability. These minimum required risks depend on how they are distributed throughout different age groups

Age	60	70	90	Source
Probability of contralateral hip fracture (model A)	4.67%	5.73%	7.11%	[16]
Probability of contralateral hip fracture (model A)	3.76%	5.54%	6.77%	[37]
Probability of contralateral hip fracture (model A)	1.42%	5.31%	13.57%	[17]
Probability of hip fracture in high risk (model B)	0.58%	3.29%	8.38%	[37]
Probability of hip fracture in high risk (model B)	0.59%	3.25%	13.37%	[17]

**Fig. 2:**
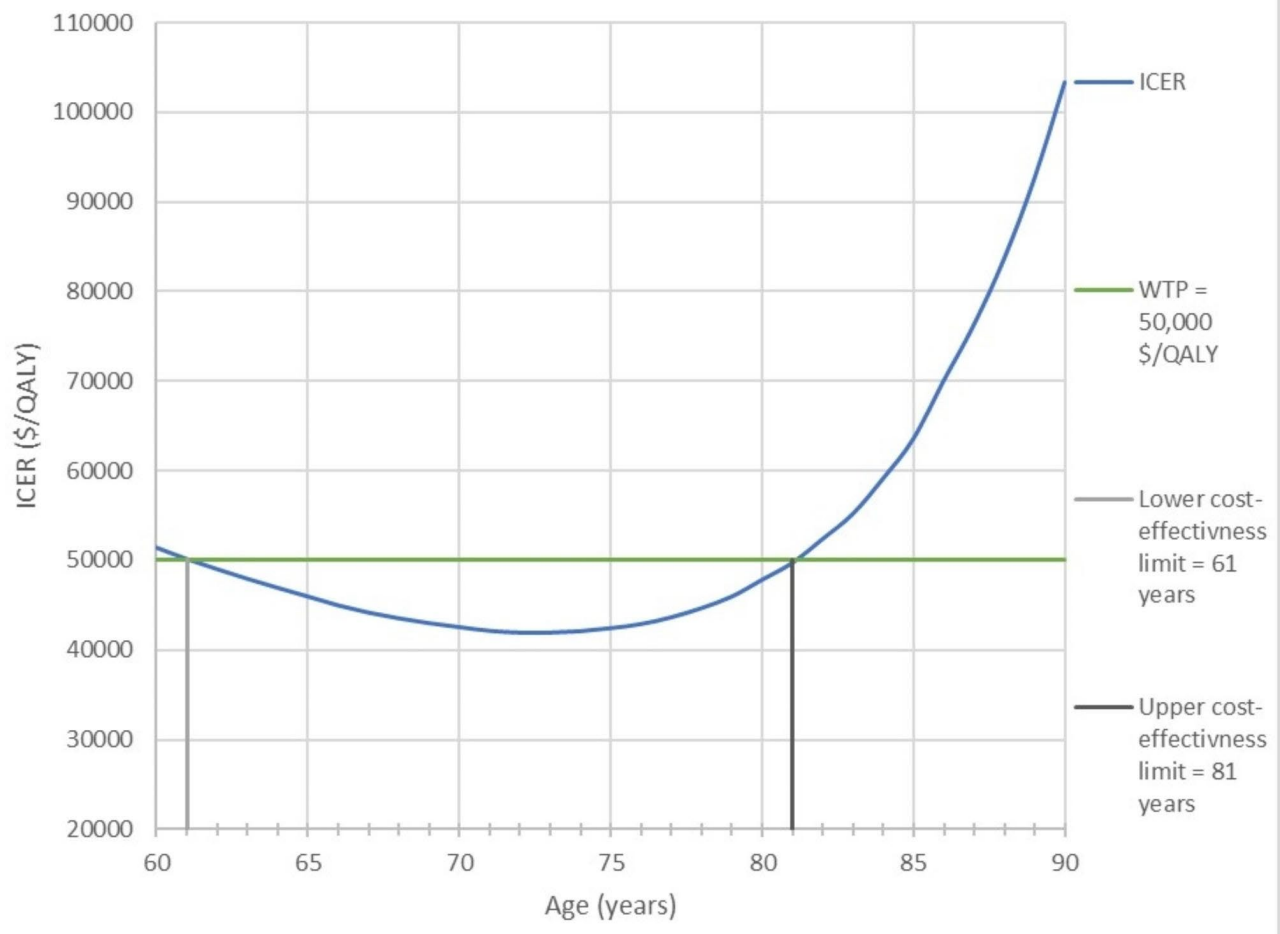
Model A: One-way worst-case scenario sensitivity analysis comparing changes in the ICER concerning age. The fracture risk used in this analysis was the lowest risk that we could obtain from the FRAX tool. The age range where secondary surgical prophylaxis is most cost-effective can be clearly seen on this figure

**Fig. 3﻿:**
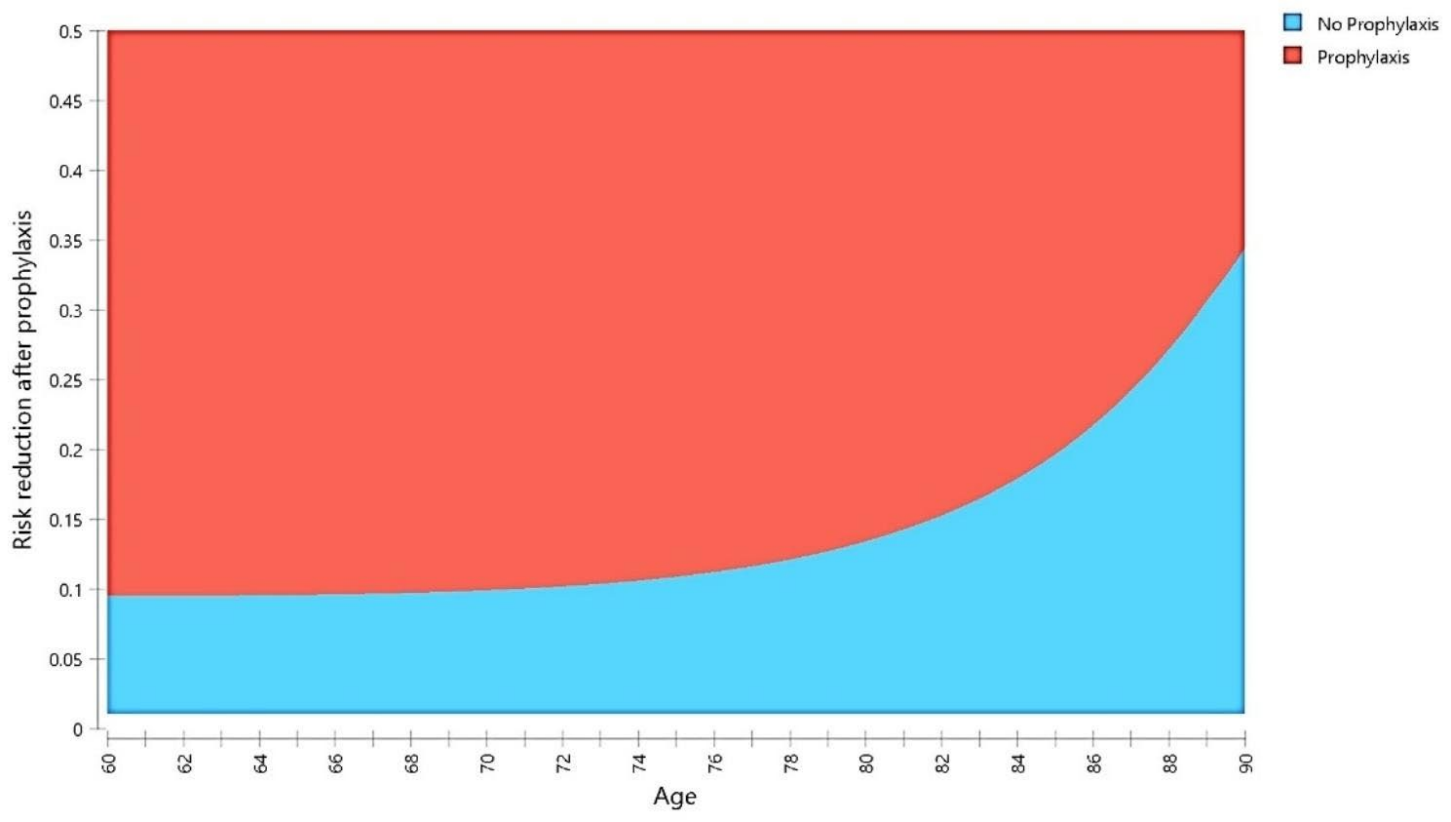
Model A: Two-way sensitivity analysis comparing the risk reduction after prophylaxis with different ages. The red area indicates that the ICER is less than $50,000/QALY, given the selected parameters. At the assumed 10% risk reduction after prophylaxis, it will be definitely cost-effective at the maximum age of 71 years

Other parameters that model A was most sensitive to were the cost of the contralateral side’s prophylaxis, the simulated cohort’s age, and fracture treatment cost. The contralateral side’s prophylaxis should cost between $1,200 and $3,700 to be definitely cost-effective in all models in the baseline analysis. When the simulated cohort was younger than 71 years old, all models were definitely cost-effective, provided that the prophylaxis-induced risk reduction was 10%. However, we performed a worst-case scenario analysis that was able to show the range of ages where prophylaxis could be most cost-effective (Fig. 2). It was carried out while ignoring the elevated fracture risk due to osteoporosis. Additionally, prophylaxis was definitely cost-effective in the baseline analysis when the estimated cost of fracture treatment varied within the defined range of $11,000 to $85,000. The two-way sensitivity analysis performed on the parameters to which the ICER was most sensitive is shown in Fig. 3. Moreover, we managed to approximate the minimum required practical age-dependent fracture risk for prophylaxis to be definitely cost-effective in both models.

### Model B

**Fig. 4:**
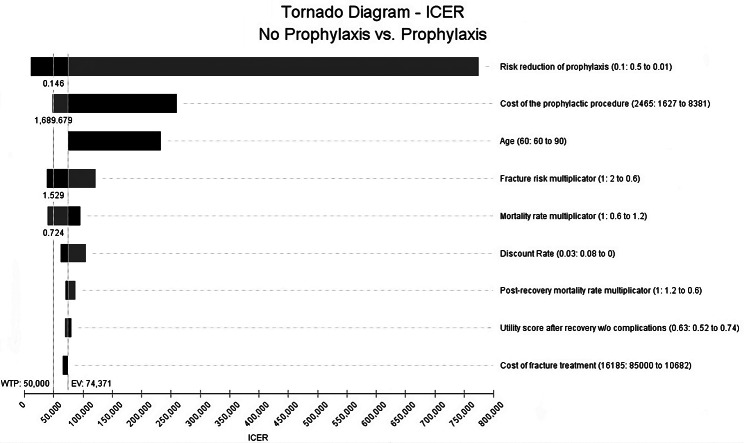
Model B: The ICER variations in terms of changes in the model parameters in the pre-defined range. Heading towards the low parameter range is represented by the bar’s grey section, while the dark section represents the high parameter range. The parameters are sorted from the most considerable effect on the ICER to the lowest. The remaining parameters that had an insignificant effect on the ICER within the defined range were removed from this diagram. The expected value (EV) line is the value where all bars are centered. It portrays the ICER for model B using the lower fracture risk. The WTP threshold line is shown as the vertical dotted line

**Fig. 5:**
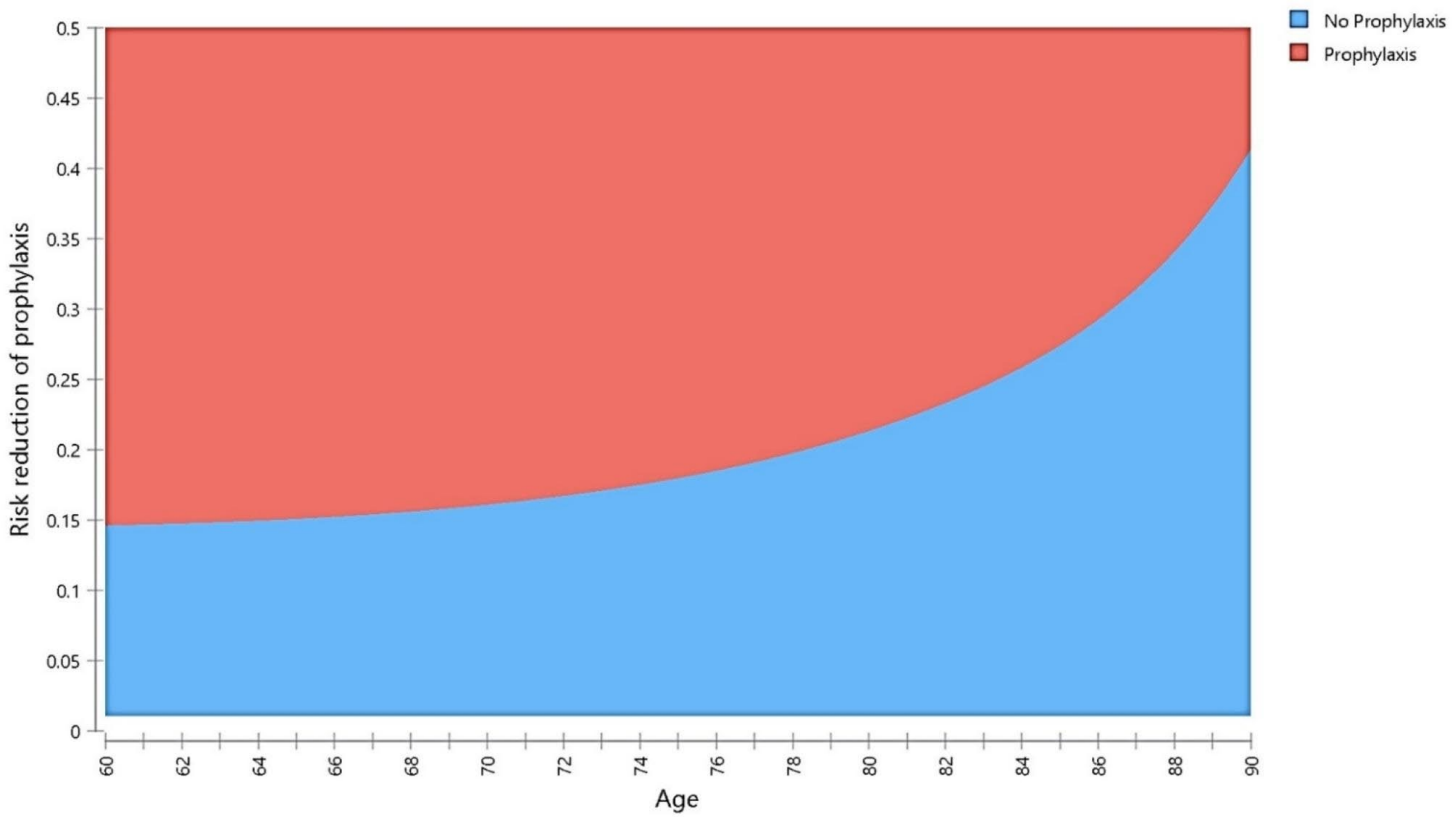
Model B: Two-way sensitivity analysis observing changes in the cost-effectiveness regarding the individual’s age and the risk reduction after prophylaxis. The red area indicates that the ICER is less than $50,000/QALY, given the selected parameters. For instance, at 15% risk reduction, the maximum age where it will be definitely cost-effective is 64 years

Depending on the fracture risk, the ICER in model B widely varied between $11,000/QALY and $74,000/QALY in the baseline analysis. For a 60-year-old individual presented with multiple risk factors, even prophylaxis that reduces the fracture risk by just 2,9% could be cost-effective. However, with the lower fracture risk in the baseline analysis, prophylaxis slightly improved QALYs (11.58 compared to 11.55 without prophylaxis) and was likely cost-effective, costing $74,400/QALY. We performed a sensitivity analysis on the lower fracture risk to evaluate the worst-case scenario. It showed that some changes in the model’s parameters within our defined ranges caused prophylaxis to be definitely cost-effective, as seen in Fig. 4. Furthermore, the two-way sensitivity analysis performed on the model with the lower fracture risk is shown in Fig. 5. Similar to model A (Fig. 3), the older the individual is, the more risk reduction is required from the prophylaxis to be cost-effective.

## Discussion

This study’s domain is utterly unprecedented since preventive orthopedic trauma surgery for hip fractures does not yet exist. Previous studies have explored the cost-effectiveness of several hip fracture preventive strategies [13, 16, 17, 45, 46], but we are not aware of any similar study investigating a mini-invasive surgical intervention such as femoroplasty. Furthermore, the study’s innovative nature exposes various uncertainties and embraces unpredictabilities. Nevertheless, this paper is meant to be the first step in investigating this unexplored field.

To a certain extent, orthopedic surgeons can recognize patients with a high risk of hip fracture. However, clear, standardized preventive procedures are limited in today’s literature. In this study, a Markov model approach was used to assess a prophylactic treatment’s cost-effectiveness during fracture operation on the contralateral side or as a preventive procedure for high-risk patients. In both cases, prophylaxis proved to be an intervention that will contribute to solving the related major socioeconomic problem due to its relatively low price compared to fracture treatment cost. The idea of simply augmenting the proximal femur with bone cement might prove revolutionary, as it does not cause any damage to the bone or the soft tissues. Accordingly, it would require very short stays in SNF and no extra rehabilitation, leading to a better overall quality of life. Even when performing worst-case scenario analysis, a substantial portion of the results resides in the cost-effective plane. Since this paper aims to help identify which patients could benefit best from prophylactic treatment, the most prominent results are the various sensitivity analysis ranges. These ranges can be categorized into three branches: risk, cost, and age.

The minimum required risk for prophylaxis to be cost-effective depends on how the risk is distributed throughout different age groups. We managed to approximate a range that sets the minimum risk for a surgeon to consider (Table 6). However, the obtained range is merely numerical and challenging for decision-makers to interpret. Further research must assign risk factors and their influence on the probability of a hip fracture. One of the most notable ranges extracted from the sensitivity analysis is the maximum allowed cost of the prophylactic surgery and the minimum required cost of fracture treatment for prophylaxis to be cost-effective. The entity of the obtained ranges lies within the predefined actual cost range. This further supports the claim mentioned in the preanalysis stating prophylaxis’s economic advantages while considering changes in quality of life.

The patient’s age is a crucial aspect to consider when deciding to perform prophylactic surgery. Thus, a worst-case scenario analysis was performed following the sensitivity analysis regarding the cohort’s age for model A while assuming a 10% relative risk reduction due to prophylaxis. Even though the worst-case scenario showed that prophylaxis is definitely cost-effective for ages between 61 and 81 years, combining all results indicates that ages 61 to 71 were the most cost-effective in all cases. Therefore, prophylactic surgery on the contralateral side for a patient aged 71 to 81 is likely cost-effective and depends on other factors, such as fracture risk and mortality rates.

In model B and most cases of model A, the cost-effectiveness of prophylaxis decreased exponentially with increasing age. This is due to the multiplication of the all-cause age-specific mortality rates with the assumed different hazard ratios following various health events, which resulted in a high death probability, reaching 100% in some cases. Additionally, an older individual with a lower life expectancy would spend fewer years in the model, decreasing the number of prevented fractures and decreasing the total QALYs gained due to prophylaxis. This raises a broad ethical discussion beyond this paper’s scope. However, we believe that when presented with limited resources, the most cost-effective decision should be made with society’s benefit as a whole in mind. The results of the deterministic sensitivity analysis are represented in the tornado diagram (Fig. 4). Despite the variation in the values, they mostly fluctuated close to the baseline analysis (EV) line, proving the reliability of the conducted evaluation.

This study has several limitations that must be addressed. First, as mentioned in the preanalysis, the risk factors’ lack of definition increased the range of the obtained results in model B, thus increasing the outcome’s vagueness. Furthermore, we did not assume the possibility of a second hip fracture for high-risk individuals despite previously mentioning the elevated risk of a hip fracture reoccurring. It was also assumed that prophylaxis was performed on only one side in model B, which is to criticize. Regarding the length of SNF and acute hospital stay, only one day was incorporated for the primary prevention strategy (model B), and no extra SNF stay was considered for the secondary prevention strategy (model A). Those lengths of stay might not be very accurate as the target groups are old and particularly frail individuals. However, there is no evidence that would suggest longer stays, given the novelty of the conducted research and that femoroplasty does not cause any damage to the bone or the soft tissues. Additionally, up to 340% of the calculated costs were assessed in the sensitivity analysis and did not affect the drawn conclusion. That indicates that even if the length of the actual SNF stay is more than 3.4 days, performing prophylaxis would still be cost-effective. A further limitation is neglecting the effect of any pharmacologic fracture prevention therapy in the calculations. Those interventions have consistently demonstrated significant potential in fragility fracture prevention [47–49]. Hence, there remains a compelling need for future research to explore the cost-effectiveness of combining surgical procedures, like femoroplasty, with such pharmacological measures.

For both models to be applicable, several real-life factors were neglected during the evaluation: (1) The elevated morbidity and risk of complications due to surgical prophylaxis, as it is an additional intervention that might necessitate repositioning of the patient, include cement injection, and extend the surgery duration. However, since surgical prophylaxis represents a theoretical intervention that has not yet been validated in clinical trials, our capacity to define its potential morbidity, complications, or prolonged surgical time is constrained. Nevertheless, the sensitivity analysis delved into possible fluctuations in post-surgery disutility and complication risks, illustrating that they did not considerably influence our main conclusions. (2) The higher morbidity of a fracture occurring on a treated femur than on a nontreated one. (3) The variation of fracture risk with the sex of the individual. (4) The major comorbidities or complications that can occur following a hip fracture by not using DRG code 480 (hip and femur procedures except major joint with major complication or comorbidity) but using DRG codes 481 and 482 (hip and femur procedures except major joint with or without a complication or comorbidity). Moreover, the assumption of linearity while extrapolating probabilities from past trends for ages above 90 years should also count as a limitation. Finally, while converting existing data on fracture risk from a 5- or 10-year probability to a 1-year probability, it was assumed that the fracture occurs at a constant rate over a particular time period.

The proposed modeling approach could also be more elaborate by considering more possible situations and adding more health states to the model. Nonetheless, that does not necessarily mean that it would cause the conclusion to be more accurate. Further research can support this study’s hypothetical deduction by increasing the accuracy and finding the probability distribution of the model parameters. By doing so, the next step could be performing a probabilistic sensitivity analysis with Monte Carlo simulation. This would pave the way to calculate the confidence intervals of the results. Additional calculations could provide a logical length for human trials regarding a new prophylactic surgery, such as femoroplasty.

In conclusion, we proposed a theoretical approach for a mathematical prediction, which surgeons should consider only as a foundation for decision-making. The created model attempts to use available literature to obtain the closest approximation of medical real-life hip fracture scenarios to examine the possible impact of a prophylaxis alternative. It was not achievable to include all related factors from a societal perspective. However, combining the acquired results with a more competent understanding of the patient’s risk factors can aid in the decision-making process.

### Electronic supplementary material

Below is the link to the electronic supplementary material.

Supplementary Material 1

## Data Availability

The data generated or analyzed during this study are included in this published article. The detailed data sets are available from the corresponding author upon reasonable request.

## Abbreviations

PMMA: Poly(methyl methacrylate)
CEA: Cost-effectiveness analysis
US$: United States dollars
QALYs: Quality-adjusted life years
ICER: Incremental cost-effectiveness ratio
WTP: Willingness to pay
WHO: World Health Organization
BMI: Body mass index
FRAX®: Fracture risk assessment tool
HCPCS: Healthcare Common Procedure Coding System
IPPS: Inpatient Prospective Payment System
DRG: Diagnosis-related group
CC: Complications and comorbidities
MCC: Major complications and comorbidities
SNF: Skilled nursing facility
EV: Expected value
