# Supplementary material for: Cost-effectiveness analysis of surgical proximal femur fracture prevention in elderly: a Markov cohort simulation model
Source: Cost Eff Resour Alloc. 2023 Oct 25;21:77. doi: 10.1186/s12962-023-00482-4 (PMC10601292; doi:10.1186/s12962-023-00482-4)
Supplement: Supplementary file 1 — Supplementary Material 1 [file 12962_2023_482_MOESM1_ESM.pdf]

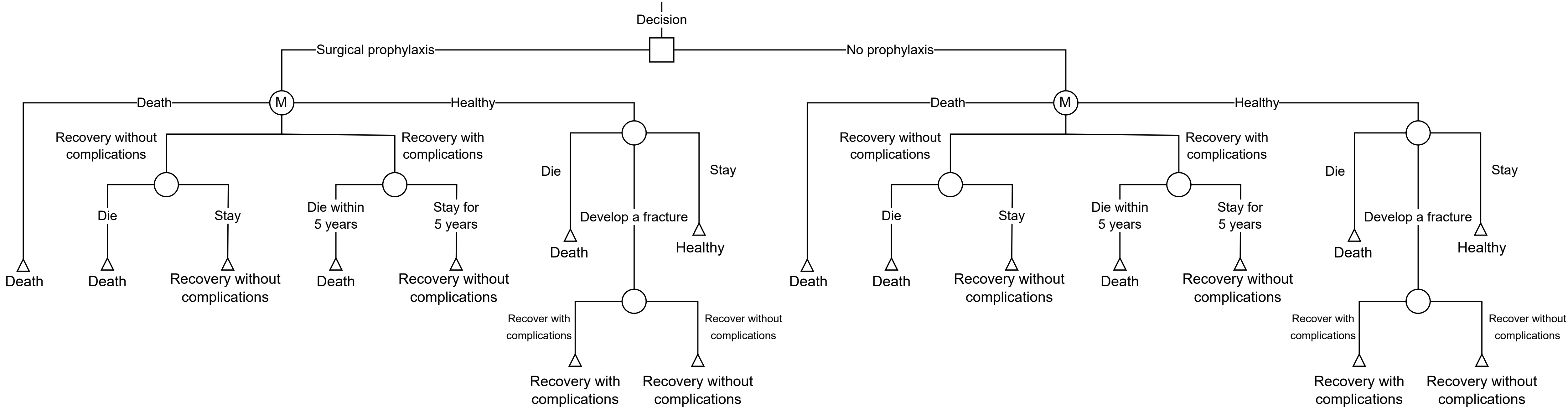

The figure shows the decision tree for the Markov model used in the analysis. It starts at a square decision node, symbolizing the choice between "Surgical prophylaxis" and "No prophylaxis." From this decision, branches extend to two Markov nodes, depicted as circles with an "M" inside, each representing a particular scenario based on the initial treatment strategy. Each line emerging from the Markov nodes signifies a distinct health state the patient might transition to within that cycle. These health states are interconnected by plain circle chance nodes, which depict the age-dependent transition probabilities between states. These probabilities, influenced by the initial treatment decision, determine the likelihood of an individual transitioning from one health state to another within a given cycle. Each branch stemming from these transitions culminates in a triangular terminal node, labelled with the health state the patient will transition into during the next cycle.
